# CNN-DDI: a learning-based method for predicting drug–drug interactions using convolution neural networks

**DOI:** 10.1186/s12859-022-04612-2

**Published:** 2022-03-07

**Authors:** Chengcheng Zhang, Yao Lu, Tianyi Zang

**Affiliations:** 1 Department of Computer Science and Technology, Harbin Institute of Technology, Harbin, China; 2 General Hospital of Heilongjiang Province Land Reclamation Bureau, Harbin, China

**Keywords:** Drug–drug interactions, Drug categories, Convolutional neural network, Multiple features combination

## Abstract

**Background:**

Drug–drug interactions (DDIs) are the reactions between drugs. They are compartmentalized into three types: synergistic, antagonistic and no reaction. As a rapidly developing technology, predicting DDIs-associated events is getting more and more attention and application in drug development and disease diagnosis fields. In this work, we study not only whether the two drugs interact, but also specific interaction types. And we propose a learning-based method using convolution neural networks to learn feature representations and predict DDIs.

**Results:**

In this paper, we proposed a novel algorithm using a CNN architecture, named CNN-DDI, to predict drug–drug interactions. First, we extract feature interactions from drug categories, targets, pathways and enzymes as feature vectors and employ the Jaccard similarity as the measurement of drugs similarity. Then, based on the representation of features, we build a new convolution neural network as the DDIs’ predictor.

**Conclusion:**

The experimental results indicate that drug categories is effective as a new feature type applied to CNN-DDI method. And using multiple features is more informative and more effective than single feature. It can be concluded that CNN-DDI has more superiority than other existing algorithms on task of predicting DDIs.

## Background

Drug–drug interactions (DDIs) mean the reactions between drugs. They are compartmentalized into three types: synergistic, antagonistic and no reaction [1–3]. The DDIs play a significant role in drug development and disease diagnosis fields, which still consumes manpower, substance sources and time [4].

Powered by advanced machine learning technology, methods of DDIs’ prediction have been evolved from traditional methods [5–7], including text mining methods and statistical methods, to machine learning methods. Furthermore, more and more studies use deep learning methods in the field of bio-informatics [8–15].

The task of predicting DDIs is vitally interrelated with similarities between drugs. The fundamental hypothesis of this task is that if drug A and drug B interact each other, causing a specific biological impact, drugs have similarity to drug A (or drug B) are possible to interact with drug B (or drug A) and causes same effect [16].

Cami et al. [17] utilized a logistic regression model to solve the DDIs’ problem. On this basis, Gottlied et al. [18] exploited more different drug–drug similarities and proposed another logistic regression model. Two similarity-based models based on drug interaction profile fingerprints were proposed [16, 19] and a heterogeneous network-assisted inference framework was introduced by Cheng et al. [20]. Some other algorithms were extended on the task of DDIs’ prediction. For instance, TMFUF [12] is based on the triple matrix factorization, DDINMF [21] is based on the semi-nonnegative matrix factorization. Three algorithms were proposed in [22], including neighbor recommender algorithm, random walk algorithm, and the matrix perturbation algorithm. Further, they proposed a novel algorithms named ‘Manifold Regularized Matrix Factorization’. In 2019, SFLLN was proposed in [23] based on linear neighborhood regularization using four types of drug features. It is a sparse feature learning ensemble method.

DeepDDI was proposed [10] to classify the DDIs’ events from DrugBank [24]. DeepDDI calculates features’ similarity and reduces features’ dimension by principal component analysis (PCA). Lee et al. [25] concentrated on concrete types of two drugs, not simply whether they interact or not. DDIMDL [11] is a multimodal deep neural network algorithm, which combines diverse drug features that predicting 65 types of DDI events.

Convolutional neural network (CNN) is a typical artificial neural network based on supervised learning, which has good performance on computer vision filed [26]. And it develops more network structures from CNN. They have been used extensively in bio-informatics [27, 28]. Many studies apply deep learning method in the task of DDIs’ prediction, and most of them choose deep neural network (DNN). But compared with deep neural network, CNN performs better in feature learning and can alleviate the degree of over-fitting effectively. Considering features selected contain noise and advantages of CNN, we decide to use CNN to solve the problem of DDIs’ prediction.

In this paper, we propose a novel algorithm based on CNN, named CNN-DDI, to learn the best combination of drug features and predict DDI-associated events. CNN-DDI method contains two parts. One part is a feature selection framework. We utilize drug categories as another feature, and choose the best combination form of drug features. The other part is a CNN-based DDI’s predictor. We utilize a new CNN to predict DDI-associated events based on features pairs selected from feature selection framework.

## Results and discussion

### Evaluation criteria

Predicting DDI’ events can be regarded as a multi-label classification problem. Therefore, the prediction results are divided into four kinds, true positive (TP), false positive (FP), true negative (TN) and false negative (FN). In addition, precision and recall criteria are common used evaluation criteria, which can evaluate the accuracy of results. Precision means in the classified positive samples, the proportion of TP samples. And recall means in all positive samples, the proportion of correct samples classified. The expressions are as follows:

1 $$precision = \frac{TP}{{TP + FP}}$$

2 $$recall = \frac{TP}{{TP + FN}}$$

Based on precision and recall, Accuracy, F1-score, area under the precision-recall curve (AUPR) and area under the ROC curve (AUC) are utilized to evaluate the performance of the algorithm.

In the study, we adopt Accuracy, F1-score, micro-averaged AUPR and micro-averaged AUC as the evaluation metrics. Micro-averaged metrics means metrics are averaged after getting the results of all classes.

### Performance

To analyze the effect of different similarity algorithms on performance of CNN-DDI, we utilize cosine similarity, Jaccard similarity and Gaussian similarity to calculate features’ similarities. Table 1 shows the experimental results of our method on three similarity measures. It can be seen that using different similarity measures exhibits similar properties. CNN-DDI is robust to these three similarity measures, so Jaccard similarity measure is used in the experiments.

**Table 1:** The experimental results of CNN-DDI on three similarity measures

Similarity	ACC	AUPR	AUC	F1	Precision	Recall
Jaccard	0.8871	0.9251	0.9980	0.7496	0.8556	0.7220
Cosine	0.8871	0.9251	0.9979	0.7492	0.8855	0.7721
Gaussian	0.8870	0.9248	0.9979	0.7489	0.8859	0.7720

To demonstrate the superiority of drug categories and influence of different combination forms, we further test the performance of CNN-DDI model with different features’ types. The experimental results are shown in Table 2. As for one feature, CNN-DDI using drug categories as the feature performs best, the AUPR score using drug categories is 0.9139, which is quite higher than the second highest score produced by drug targets (the value is 0.8470). Similarly, using drug categories achieves the highest scores of other five evaluation metrics. So the drug category is effective as a new feature type applied to CNN-DDI method. On the whole, using multiple features is informative and helps CNN-DDI perform better than single feature. The combination of four features has the highest AUPR score (the value is 0.9251) in all combinations. Thus it can be proved that every feature improves the performance of CNN-DDI to a certain extend.

**Table 2:** Results of CNN-DDI using different features

Feature	ACC	AUPR	AUC	F1	Precision	Recall
T	0.7915	0.8470	0.9953	0.6099	0.6932	0.5716
P	0.7820	0.8381	0.9952	0.5805	0.6822	0.5364
E	0.6580	0.7098	0.9897	0.3344	0.4419	0.2957
C	0.8702	0.9139	0.9966	0.7421	0.7994	0.7125
T + P	0.8227	0.8898	0.9969	0.6778	0.7589	0.6375
T + E	0.8242	0.8712	0.9956	0.6360	0.7373	0.5849
T + C	0.8792	0.9185	0.9960	0.7627	0.8167	0.7405
P + E	0.8255	0.8747	0.9958	0.6227	0.7130	0.5781
P + C	0.8796	0.9179	0.9961	0.7440	0.7955	0.7485
E + C	0.8496	0.8895	0.9948	0.6928	0.7726	0.6488
T + P + E	0.8243	0.8690	0.9947	0.6489	0.7332	0.6063
T + P + C	0.8797	0.9199	0.9960	0.7490	0.8164	0.7232
T + E + C	0.8539	0.8899	0.9933	0.6938	0.7726	0.6539
P + E + C	0.8559	0.8919	09,939	0.6845	0.7575	0.6485
T + P + E + C	**0.8871**	**0.9251**	**0.9980**	**0.7496**	**0.8556**	**0.7220**

The bold values indicate the result of CNN_DDI with four types of features. So it can be concluded that the drug category is effective as a new feature type and multiple features can imporve the performanced of CNN-DDI

### Comparison experiments

We evaluate the effectiveness of our algorithm and four state-of-art algorithms. The four algorithms are random forest (RF), gradient boosting decision tree (GBDT), logistic regression (LR) and K-nearest neighbor (KNN). We measure feature similarities in the same manner. In the experiment, we set the decision tress number of RF to be 100 and the neighbor number of KNN to be 4.

Table 3 shows CNN-DDI algorithm has better performance than other four methods in these 6 accuracy assessments. The score of ACC is 0.8871, it is better than the score of GBDT, RF, KNN and LR (0.8327, 0.7837, 0.7581 and 0.7558 respectively). And other evaluation metrics achieved by CNN-DDI are 0.9251, 0.9980, 0.7496, 0.8556 and 0.7220, respectively, which are significantly higher than the sores of other methods. The LR algorithm gets the worst performance, whose scores are 0.7558, 0.8087, 0.9950, 0.3894, 0.5617 and 0.3331, respectively. Compared with GBDT, which gets the second best performance, the ACC score is 0.8871, increased by 6.53%. And the score of AUPR is 0.9251, increased by 4.79%, all of other evaluation metrics have been improved in varying degrees.

**Table 3:** Results of CNN-DDI and other state-of-art models

Algorithm	ACC	AUPR	AUC	F1	Precision	Recall
CNN-DDI	0.8871	0.9251	0.9980	0.7496	0.8556	0.7220
GBDT	0.8327	0.8828	0.9970	0.6730	0.7817	0.6133
RF	0.7837	0.8446	0.9959	0.5167	0.6973	0.4444
KNN	0.7581	0.8166	0.9881	0.6250	0.7562	0.5596
LR	0.7558	0.8087	0.9950	0.3894	0.5617	0.3331

And we compare our algorithm with DDIMDL. Considering DDIMDL using different features, we retrain DDIMDL model with features selected by CNN-DDI. As shown in Table 4, DDIMDL represents the original algorithm proposed by original paper [11]. DDIMDL* represents DDIMDL with features selected by CNN-DDI. It can be concluded that the drug category is effective as a new feature type, and CNN-DDI still performs better than DDIMDL in the case of using the same features.

**Table 4:** Comparison of CNN-DDI with DDIMDL

Algorithm	ACC	AUPR	AUC	F1	Precision	Recall
CNN-DDI	0.8871	0.9251	0.9980	0.7496	0.8556	0.7220
DDIMDL	0.8852	0.9208	0.9976	0.7585	0.8471	0.7182
DDIMDL*	0.8865	0.9230	0.9976	0.7559	0.8513	0.7204

The single asterisk represent DDIMDL with features selected by our method. It can be concluded that the drug category is effective as a new feature type

## Conclusions

In the work, we proposed a novel semi-supervised algorithm using a CNN architecture, named CNN-DDI, to predict drug–drug interactions. First, we extract feature interactions from drug categories, targets, pathways and enzymes as feature vectors. Then, based on the representation of feature, we proposed a new convolution neural network as the predictor of DDIs-associated events. The predictor consists of five convolutional layers, two full-connected layers and a softmax layer based on CNN.

To demonstrate the performance of our method, we compare it with other start-of-the-art methods. The evaluation shows our method, CNN-DDI, has better performance than other existing state-of-art measures. Meanwhile, we discuss the contribution of combinational features and each single feature. Overall, CNN-DDI has more advantages on predicting DDIs’ events. In consideration of consuming longer time, we will try to improve the efficiency of CNN-DDI in the future.

## Methods

**Fig. 1:**
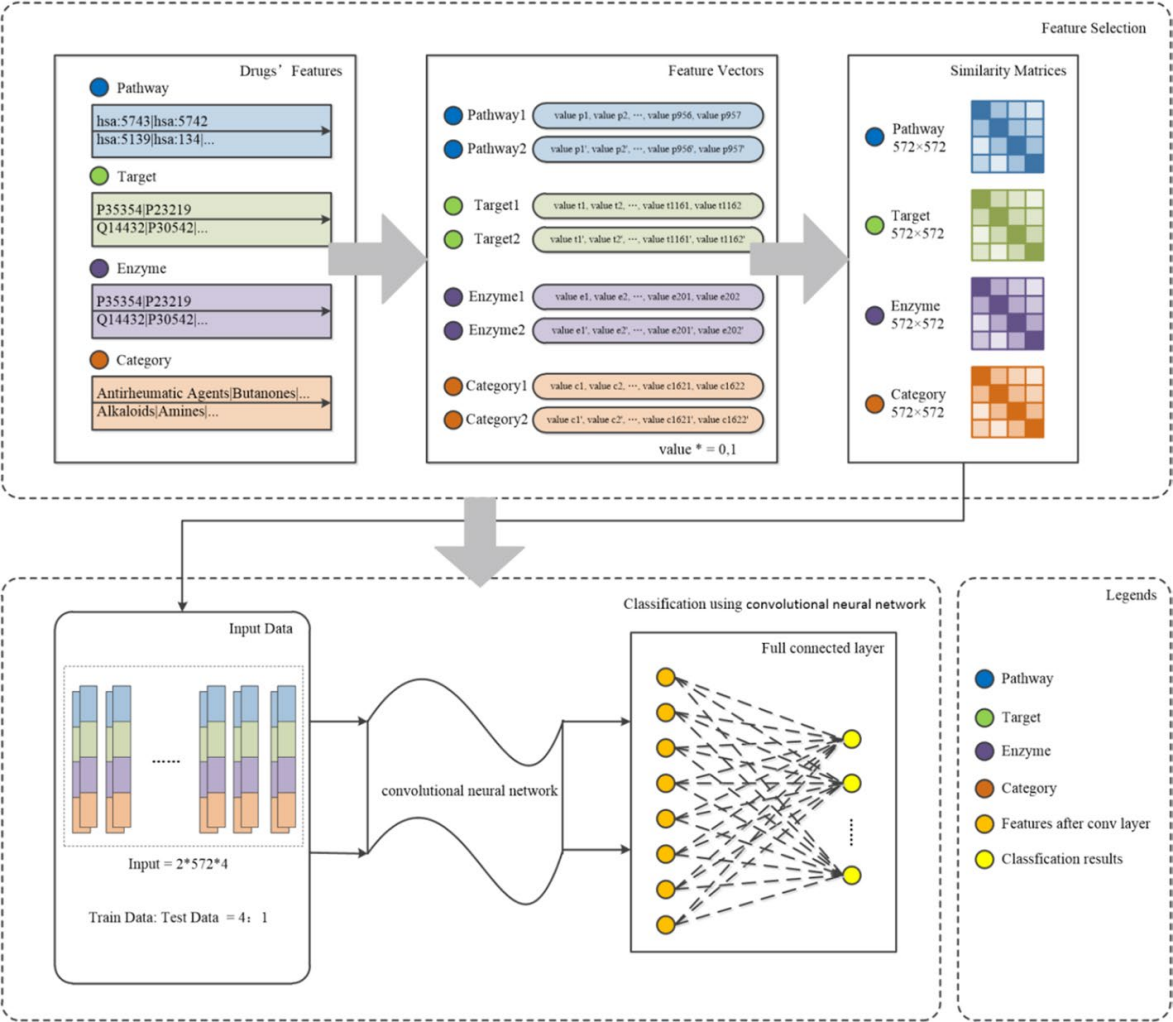
The framework of CNN-DDI algorithm.The algorithm mainly contain two parts, combinational features selection module and CNN-based prediction module. (1)Firstly, features vectors are selected from feature selection module using the four types of features. We encode features and generate binary vectors, each value of the vector represents whether the component exists. Then we calculate Jaccard similarity to measure the correlation between drugs. In this way, we get features vectors as the input of the prediction module.Secondly, features vectors are inputted into prediction module. The prediction module based on CNN consists of convolutional layers, full-connecteed layers and a softmax layer.Convolutional layers can enhance the ability of learning deep characteristics. Through the DDIs’ predictor, we get the probabilities of all DDIs-associated events’ types and select the event with the highest probability

We propose a novel method called CNN-DDI to predict DDI-associated events. The method mainly contain two parts, combinational features selection module and CNN-based prediction module. As shown in Fig. 1, we combine four drug features and obtain a low dimensional as the CNN model inputs. Then a deep CNN model is built to calculate the probability of DDIs’ types. In this section, we will thoroughly expound the structure and principle of CNN-DDI.

### Data collection

DDIMDL proposed a data set that classifying DDIs’ events into 65 types, not simply focusing on whether they interact or not. The data set includes 572 drugs and 74,528 DDIs-associated events collected from DrugBank. Which is a manually collected data source that provides drugs comprehensive information and unified syntax in describing DDIs.

To extend the information of DDIMDL, we extract drugs categories from DrugBank. 572 drugs have 1622 types of categories in DDIMDL.

In our paper, cross validation is utilized to demonstrate the effectiveness of our method. We set the fold number of cross validation is 5. In our experiments, we randomly divide the data set into five subsets, choose four subsets as the train set and another one as the test set. We test on the data set five times following the above steps, and the final result is the average of multiple results.

### CNN-DDI algorithm

#### Drug–drug similarity

There are three common similarity measures, Jaccard similarity, cosine similarity and Gaussian similarity. To better measure the drug feature vectors’ similarity, we analyze the difference of measures’ results. Jaccard similarity calculates the intersection of components and the union. Gaussian similarity utilizes the Gaussian kernel function. And cosine similarity is used to calculate the cosine between two vectors in an inner product space [29].

Jaccard similarity can be calculated as follows:

3 $$\begin{aligned} Sim_{J} \left( {x_{i} ,x_{j} } \right) & = Sim_{J} \left( {X,Y} \right) = \frac{{\left| {X \cap Y} \right|}}{{\left| {X \cup Y} \right|}} \\ & = \frac{{M_{11} }}{{M_{01} + M_{10} + M_{11} }} \\ \end{aligned}$$

where *x*_*i*_ and *x*_*j*_ are feature vectors of two drugs, X and Y are the vector sets respectively. $\left| {X \cup Y} \right|$ represents the union of X and Y, $\left| {X \cap Y} \right|$ represents the intersection. Further, M represents the number of elements. Subscript 11 means the elements where *x*_*i*_ and *x*_*j*_ are 1, 01 means elements where *x*_*i*_ is 0 and *x*_*j*_ is 1, 10 means elements where *x*_*i*_ is 1 and *x*_*j*_ is 0.

Cosine similarity can be calculated as follows:

4 $$Sim_{c} \left( {x_{i} ,x_{j} } \right) = \frac{{x_{i} \cdot x_{j} }}{{x_{i} x_{j} }}$$

where $|| \cdot ||$ represents the Euclidean norm.

Gaussian similarity can be calculated as follows:

5 $$Sim_{G} \left( {x_{i} , x_{j} } \right) = \exp \left( { - \gamma x_{i} - x_{j}^{2} } \right)$$

where $\gamma$ represents hyper parameters. And $\gamma = 1/\left( {\mathop \sum \limits_{i = 1}^{n} \left| {x_{i} } \right|/n} \right)$.

#### Feature selection module

Firstly, we evaluate the similarity between two drugs. The feature selection includes two steps: (1) calculating the similarity scores to evaluate correlation between drugs. (2) Generating feature vectors as the input to the prediction module.

The drugs’ feature can be represented as a binary vector, the value is 1 or 0. Value 1 means presence of components, value 0 means absence. For instance, the data set has 1622 types of categories. So the categories can be expressed as a 1622-dimensional bit vector, the value means that the drug belongs to the category or not. Similarly, we can extract four binary feature vectors from one drug corresponding four features. Then we calculate the similarity between two drugs’ feature vectors by similarity measures. By this means, similarity matrices are generated as $S = \left( {s_{ij} } \right)$, where the value of ${\text{s}}_{{{\text{ij}}}}$ is from 0 to 1. The closer the value is to 1, the higher the similar degree of drugs.

#### Prediction module using convolutional neural network

As shown in Fig. 1, CNN-based prediction module is the important part to predict DDIs’ events. Features selected from selection module are input vectors into the prediction module. Considering features selected contain noise and advantages of CNN, we decide to use CNN in the prediction module.

CNN is widely used and performs well on computer vision, like image classification, image detection and image segmentation. And powered by advanced deep learning technology, more and more studies have explored its application in bio-informatics field [30]. Compared with the pure deep neural network, CNN has the following advantages: (1) the convolutional layer has less parameters by using connections’ sparsity and parameters sharing. (2) The convolutional layer extracts information from global features and local features. On the task of DDIs’ prediction, Results of classification are strongly related to not only global drug features but also part of features combination. So it can enhance the capability of features learn. Consequently, in this article, we apply CNN as the supervised model for distilling integrated features information to predict DDIs.

**Fig. 2:**
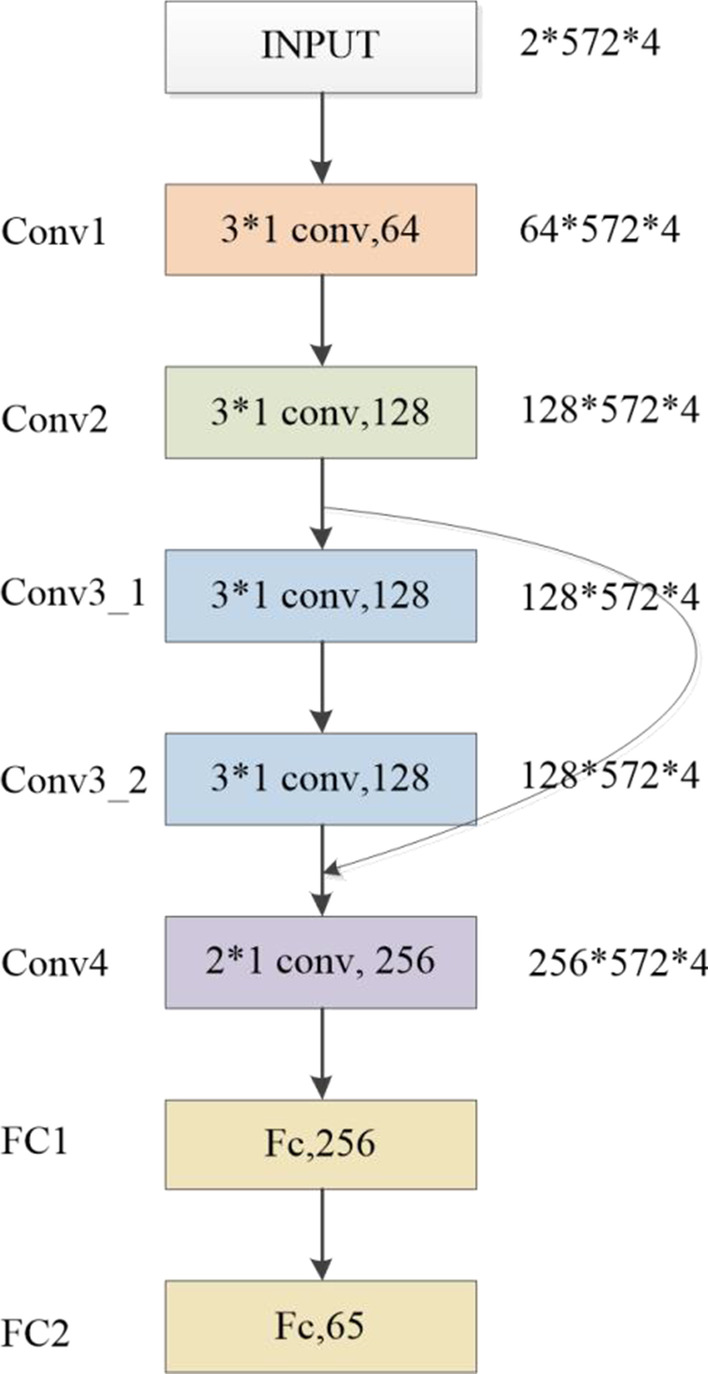
The structure of prediction model

The structure of prediction model is shown in Fig. 2. The prediction model based on CNN includes five convolutional layers, two full-connected layers and a softmax layer. Among them, convolutional layers are mainly responsible for subspace feature extraction from the input vectors. Table 5 shows the specific configuration. The kernel size of each convolutional layer is same (3 × 1), and the filters’ number is increasing layer-by-layer.

**Table 5:** The convolution layers of CNN-DDI

Layer name	number of filters	Kernel size	Output shape
Conv1	64	3 × 1	(64, 572, 4)
Conv2	128	3 × 1	(128, 572, 4)
Conv3_1	128	3 × 1	(128, 572, 4)
Conv3_2	128	3 × 1	(128, 572, 4)
Conv4	256	3 × 1	(256, 572, 4)

**Fig. 3:**
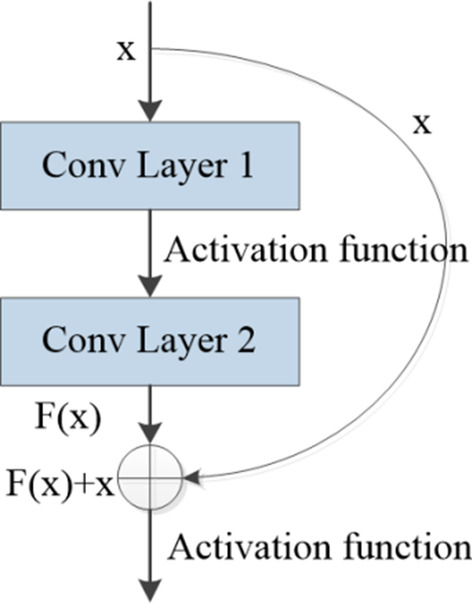
The structure of residual block

In addition, we add a residual block [31] to build one short connection between two layers. Figure 3 shows the structure of residual block. The output of residual block is expressed as follows:

6 $$y = F\left( x \right) + x = W_{2} \left[ {\sigma_{1} \left( {W_{1} x + b_{1} } \right)} \right] + b_{2} + x$$

where *x* is the input vectors, *y* is the output vectors. *W*_1_, *W*_2_ are the weight vectors of two layers, *b*_1_, *b*_2_ are the biases, and $\sigma_{1}$ is the activation function of first layer.

The residual block strengthen the correlation of multi-layer features. The short connection’s input vectors and output vectors must have the same dimensions, and the stacked convolutional layers’ output vectors are added together. It should be noted that no additional parameters are added in the residual block.

The output of each convolutional layer is passed through an activation function that enhances positive vectors and inhibits negative vectors from previous layer. In the paper, the activation function we use is Leaky ReLU. Compare with other activations, ReLU can increase feature sparsity and decrease the possibility of vanishing gradient. The expression is as follows:

7 $$LeakyRelu \left( x \right) = \left\{ {\begin{array}{*{20}l} {x, } \hfill & {x \ge 0} \hfill \\ {ax,} \hfill & {x < 0} \hfill \\ \end{array} } \right.$$

where a represents hyper-parameters, a is set 0.2.

There are two full-connected layers after convolutional layers. The first full-connected layer has 267 hidden units and the second has 65 hidden units. Considering predicting DDI’s events is a classification task, softmax function is used as the activation of the last full-connected layer. So the loss function of the prediction module is as follows:

8 $${\text{Loss}} = { } - \mathop \sum \limits_{i = 1}^{K} y_{i} log\left( {p_{i} } \right)$$

where K represents the number of events’ types, *y*_*i*_ represents the true value, 0 or 1.

#### The CNN-DDI algorithm

The algorithm mainly contain two parts, combinational features selection module and CNN-based prediction module. The pseudocode of CNN-DDI is shown in Algorithm 1.
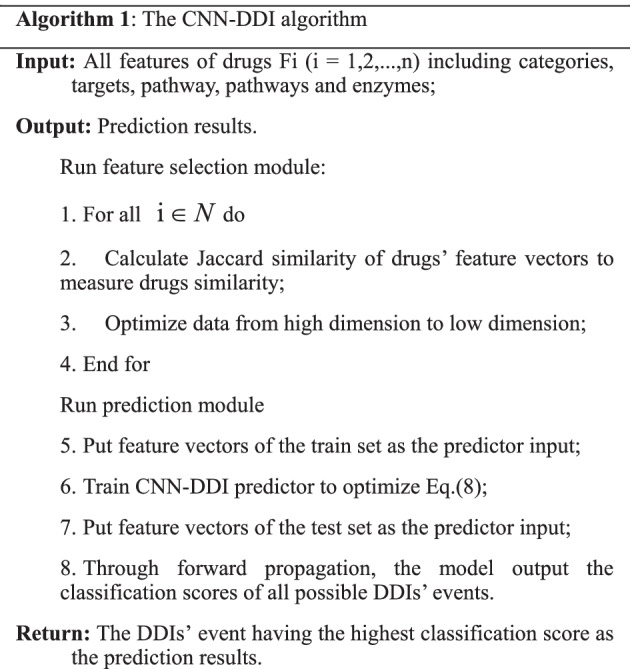


## Data Availability

The datasets generated and/or analyzed during the current study are available in the drugbank repository and DDIMDL repository. https://go.drugbank.com/https://github.com/YifanDengWHU/DDIMDL.

## Abbreviations

DDIs: Drug–drug interactions
CNN: Convolutional neural network
DNN: Deep neural network
AUPR: Area under the precision-recall curve
AUC: Area under the ROC curve
RF: Random forest
GBDT: Gradient boosting decision tree
LR: Logistic regression
KNN: K-nearest neighbor
PCA: Principal component analysis
